# Combining CBT and sertraline does not enhance outcomes for anxious youth: a double-blind randomised controlled trial

**DOI:** 10.1017/S0033291721003329

**Published:** 2023-04

**Authors:** Jennifer L. Hudson, Lauren F. McLellan, Valsamma Eapen, Ronald M. Rapee, Viviana Wuthrich, Heidi J. Lyneham

**Affiliations:** 1 Black Dog Institute, University of New South Wales, Randwick, NSW 2031, Australia; 2 Department of Psychology, Centre for Emotional Health, Macquarie University, Sydney, NSW 2109, Australia; 3 Academic Unit of Infant Child and Adolescent Psychiatry South West Sydney (AUCS), University of New South Wales, Sydney, NSW 2052, Australia

**Keywords:** CBT, child anxiety, SSRI, treatment

## Abstract

**Background:**

Anxiety disorders are the most prevalent mental disorder in children and young people. Developing effective therapy for these children is critical to reduce mental disorders across the lifespan. The study aimed to evaluate the efficacy of combining cognitive behavioural therapy (CBT) and sertraline (SERT) in the treatment of anxiety in youth, using a double-blind randomised control trial design.

**Methods:**

Ninety-nine youth (ages 7–15 years) with an anxiety disorder were randomly allocated to either individual (CBT) and SERT or individual CBT and pill placebo and assessed again immediately and 6 months after treatment.

**Results:**

There were no significant differences between conditions in remission of primary anxiety disorder or all anxiety disorders. Furthermore, there were no significant differences in rates of change in diagnostic severity, parent-reported anxiety symptoms, child-reported anxiety symptoms or life interference due to anxiety.

**Conclusions:**

The efficacy of CBT for children and adolescents with anxiety disorders is not significantly enhanced by combination with a short-term course of anti-depressants over and above the combined effects of pill placebo.

Anxiety disorders are the most prevalent psychiatric disorders in children and adolescents and predict increased risk for mental disorders across the lifespan (Rapee, Schniering, & Hudson, 2009). Diagnostic remission following mono-treatments for paediatric anxiety disorders using either cognitive behavioural therapy (CBT) or anti-depressant medication (particularly selective serotonin reuptake inhibitors – SSRIs) are between 50% and 60% (Ipser, Stein, Hawkridge, & Hoppe, 2009; James, Reardon, Soler, James, & Creswell, 2020). Although these results are favourable, the consequences for the 40–50% of children who do not remit following treatment are serious. Children who are not successfully treated show a significantly greater long-term risk of suicide and depression compared to those children who respond favourably to treatment (Wolk, Kendall, & Beidas, 2015).

In a large multi-site study, Walkup and colleagues were the first to evaluate the efficacy of combining psychological and pharmacological treatments in an effort to improve outcomes for anxious children (Walkup et al., 2008). The results showed that CBT + SSRI [i.e. sertraline (SERT)] produced superior outcomes to CBT alone, SSRI alone or placebo. Combining treatments produced more than 20–25% greater improvement than either treatment alone. At the follow-up, combined treatment continued to show enhanced outcomes (Piacentini et al., 2014). Although a fifth arm of this study was not feasible, a significant limitation of this study was the absence of a condition that included CBT and a pill placebo. Families allocated to combined therapy received a greater dose of therapy (including visits with both a psychologist and a psychiatrist) and were aware they were receiving the ‘best’ dose, markedly increasing the expectancy effects associated with this condition. Although expectancy effects are present for all treatments, the impact of expectancy on response to pharmacological treatments is well established (Colloca & Benedetti, 2005). Expectancy effects cannot be ruled out as a plausible alternative explanation for the significantly better outcome following combination treatment. Given the dramatic increase in apparent efficacy of the combined treatment, this research needs to be replicated using a more rigorous design. To date, two small randomised controlled trials using children with obsessive compulsive disorder (OCD) and post-traumatic stress disorder (PTSD) have employed a CBT and pill placebo condition to adequately control for expectancy effects (Cohen, Mannarino, Perel, & Staron, 2007; Storch et al., 2013). Both studies failed to detect significant differences between conditions involving CBT + active medication and CBT + placebo.

The current study aimed to evaluate the efficacy of combination treatment for youth anxiety using a double-blind randomised control trial design. We hypothesised that combining CBT and an antidepressant (CBT + SERT) would lead to superior outcomes compared to CBT and a pill placebo (a medication that contains no active ingredients, i.e. rice flour). We expected that the CBT + SERT condition would produce greater diagnostic change (greater remission of primary and all anxiety diagnoses and greater global improvement), symptom change (greater reduction in primary care-giver and child-reported anxiety and internalising symptoms) and improvements in life interference caused by anxiety disorders. The primary outcome is remission of the primary anxiety disorder at post.

## Method

### Participants

**Fig. 1.**
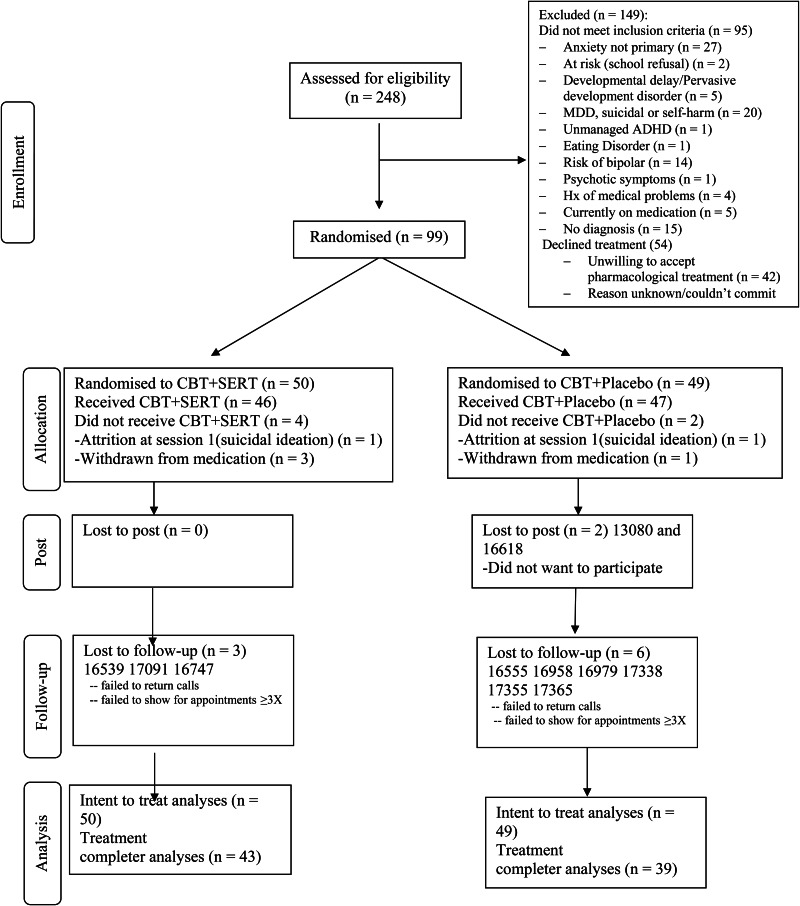
Consort diagram of participants through the study.

Participants in the study were 99 children aged 7–15 years (*M* = 9.59, s.d. = 2.05; 54 boys) meeting criteria for a primary anxiety disorder, OCD or PTSD according to DSM-5 (American Psychiatric Association, 1994). Anxiety disorders were assessed using a version of the Anxiety Disorders Interview Schedule for DSM-IV, Parent and Child Versions (ADIS-IV-C/P; Silverman and Albano, 1996) that was modified by the research team to identify diagnoses consistent with DSM-5. Participants were excluded if they met any of the following criteria: receiving concurrent pharmacological therapy [other than a stable dose of stimulant medication for attention deficit hyperactivity disorder (ADHD)]; non-response to two previous trials of SSRIs; receiving concurrent psychological treatment; current major depressive disorder, suicidal and/or are self-harming; at-risk due to abuse, neglect or extended school refusal; significant learning delays that prevent mainstream class placement (intellectual disabilities); autism or related disorders (developmental disorders); significant hyperactive behaviour that is unmanaged (i.e. ADHD); eating disorder; substance use disorder; risk of bipolar disorder (via family history); psychotic symptoms; participants with a history or current heart, kidney, liver issues, diabetes, glaucoma or epilepsy; are pregnant; inpatient; have limited English literacy. The primary diagnoses were as follows: generalised anxiety disorder (GAD) (48.5%), social anxiety disorder (23.2%), separation anxiety disorder (SAD) (14.1%), specific phobia (5%), panic disorder (2%), OCD (3%), PTSD (1%) and other specified anxiety disorder (3%). Figure 1 presents the flow of participants through the study.

### Study design and implementation

Using multi-method, multi-informant assessment of children with a range of anxiety disorders, participants were randomised to receive either a 12 week CBT + pill placebo or 12 week CBT + SSRI intervention. Diagnostic assessments and treatment were conducted at Macquarie University (recruitment: 05/2014 until 09/2015; follow-up: 3/2015 until 7/2016). The study followed CONSORT guidelines and was registered with the Australian and New Zealand Clinical Trials Register (ACTRN12613001059752). The trial was also registered with the Australian Government Therapeutic Goods Administration (TGA) Clinical Trial Notification system given the off-label use of medication. The trial was considered off-label because, although SERT is approved by the TGA for use with children and adolescents with a primary anxiety diagnosis of OCD, it is considered off-label for children with primary anxiety disorders. The trial was approved by Human Ethics Committee at Macquarie University. Parents provided written consent and children verbal assent or written consent for older children. The trial protocol is available from the authors.

### Randomisation and masking

Children were randomly allocated according to a list through a random number generator (RR). The study pharmacist dispensed medication according to the allocation order. Following treatment, an assessment session (including a structured diagnostic interview and parent- and child-reported questionnaires) occurred in week 13 and then again 6 months following the completion of treatment. Parents, children, diagnosticians, therapists, psychiatrists, supervisors, clinic staff and the project manager were masked to condition. After the 6-month assessment, families were made aware of the treatment condition to which they had been allocated.

### Outcome measures

The primary outcome measure was the remission of anxiety disorders: remission of the primary anxiety disorder (most interfering) as well as remission of all anxiety diagnoses. Remission was defined as a clinical severity rating (CSR) of below 4. All children and parents were interviewed by an independent diagnostician using a modified version of the ADIS-IV-C/P (Silverman & Albano, 1996). Diagnoses and CSRs (on a scale of 0–8) were assigned by graduate students in clinical psychology based on composite parent and child report. Interviews (10%) were double coded for reliability purposes demonstrating excellent agreement for primary disorder (80%) and the presence of the following disorders: SAD (100%), GAD (100%), social anxiety disorder (90%) and specific phobia (100%). Reliability for the CSRs was also excellent for the primary and secondary diagnosis: intraclass correlation (ICC) (2.1) = 0.84.

Secondary outcome measures included: (i) change in the CSR of the primary anxiety disorder; (ii) Clinical Global Impressions scale assessed by the independent diagnostician measured improvement of anxiety diagnoses (no change, minimal change, improved, very much improved); (iii) The Spence Children's Anxiety Scale (SCAS) (Spence, 1998) to assess child and primary care-giver-reported anxiety symptoms; (iv) the Short Mood and Feelings Questionnaire (SMFQ; Angold et al., 1995) to assess child and primary care-giver-reported depressive symptoms and (v) Child Anxiety Life Interference Scale (Lyneham et al., 2013) to assess parent- and child-reported life interference and impairment associated with the child's anxiety. Mothers were the primary care-givers in 90.9% of participants.

### Interventions

Children and parents received 10 sessions of manualised CBT over 12 weeks. The manualised programme, Cool Kids (Rapee et al., 2006), is designed for the management of a range of childhood anxiety disorders and includes affect recognition, cognitive restructuring, child management, social skills training, assertiveness and gradual exposure. Seven sessions (4–10) are devoted to designing and evaluating the child's weekly gradual exposure tasks. Therapists were graduate clinical psychology students trained in the Cool Kids treatment programme and received weekly supervision from the study authors. An independent rater examined 100 therapy sessions (10%) and assessed the degree to which the therapist adhered to the treatment protocol. There were no instances of therapy breaches. Homework was appropriately scheduled and reviewed in 100% of sessions (excluding the first and last session). Gradual exposure was introduced in session 3 and addressed in 100% of all evaluated sessions between sessions 3 and 10.

Weekly pill counts and daily medication diaries were used to facilitate and document medication adherence. Parents completed the SSRI side effects checklist (Sharp & Hellings, 2006) and the Spence Children's Anxiety Scale (Spence, 1998) each week to monitor symptoms and side effects. The child and parents also received three psychiatrist visits at weeks 1, 7 and 13. To minimise the risk of adverse events and increase tolerance to the medication, dosage for all children started at 25 mg for 7 days and was then increased by 25 mg to 50 mg/day. The 50 mg/day dosage was maintained for a 2-week initial dosage stabilisation period according to tolerability and response. At the mid-treatment point, dosage was increased beyond the initial maximum of 50 mg/day up to a maximum of 150 mg, if anxiety symptoms continued to be impairing and medication was tolerated. At the end of treatment, dosage was gradually decreased to 25 mg for a week followed by 25 mg on alternate days for a week after the final session/post assessment.

Parents and children also completed a six item Credibility/Expectancies Questionnaire to assess credibility and expectancy, separately for the CBT aspect of treatment and the medication component (Nock & Ferriter, 2005) before and immediately post treatment. Items were rated on a 1–9 scale or a 0–100% scale (recoded to a 9 point scale as per Nock & Ferriter, 2005). A total score for both parent and child report was calculated for each treatment component, as well as subscales for credibility and expectancy.

### Statistical analysis

Statistical Program for the Social Sciences (SPSS version 24.0) was used to conduct all analyses. The study enrolled 99 patients provided 85% power to detect a treatment difference (*w* = 0.3) at an alpha level of 0.05 (GPower). Intention to treat analyses was conducted and reported using all available data. Analyses utilising the intent-to-treat and completer samples produced comparable results that did not alter the significance of the test, hence the intent to treat (ITT) analyses are conducted.

The proportion of participants who no longer met criteria for: (i) the primary anxiety diagnosis and (ii) any anxiety diagnosis, at post and 6-month follow-up in the two conditions was examined using chi-squared tests of independence. Mixed models containing random factors for subject, and fixed effects for condition and time (and their two-way interaction) were fitted to measures of child- and mother-reported symptoms, and diagnostic severity. Effect sizes were calculated using the estimated marginal means from the mixed models.

## Results

### Dosage adherence

Based on pill counts and medication diaries, children and families showed excellent adherence to the medication treatment protocol (CBT + SERT: 92.4%; CBT + PBO: 95.67%). The mean daily dosage at the final psychiatrist visit was 50.53 mg for the CBT + SERT condition (range = 25–100 mg) and 50.00 mg for the CBT + PBO (range = 25–75 mg).^†^^1^

### Adverse effects

Four children (CBT + SERT = 3; CBT + PBO = 1) were withdrawn from medication due to increased symptoms or parental concerns around side effects. Table 1 provides a list of the moderate to severe side effects reported across the two conditions after medication commenced. There were no significant differences in weekly symptoms reported across the two conditions with the exception of two items. Children in the CBT + PBO condition reported significantly greater mean irritability symptoms, χ^2^(1, *N* = 97) = 4.98, *p* = 0.026, and emotional outbursts, χ^2^(1, *N* = 97) = 4.67, *p* = 0.031. These differences in irritability and emotional outbursts were not present in the week prior to the commencement of medication (*p*s > 0.05). A complete list of mild, moderate and severe side effects is reported in the online Supplementary materials (Table S1). There were no significant differences in reported adverse symptoms (mild to severe) between the two conditions (*p*s > 0.05).

**Table 1.** Moderate to severe adverse effects reported using the SSRI Side Effects Scale across the two conditions

	CBT + PBO (*n* = 48)	CBT + SERT (*n* = 49)
1. Tremors	1 (2.1)	3 (6.1)
2. Fever	4 (8.3)	2 (4.1)
3. Headache	7 (14.6)	5 (10.2)
4. Dizziness	2 (4.2)	3 (6.1)
5. Body ache	4 (12.5)	2 (4.1)
6. Weight loss/decreased appetite	1 (2.1)	2 (4.1)
7. Stomach pain	4 (10.4)	10 (20.4)
8. Gastric distress	1 (6.3)	6 (12.2)
9. Constipation	3 (6.3)	2 (4.1)
10. Diarrhoea	5 (10.4)	5 (10.4)
11. Nausea	5 (10.4)	8 (16.3)
12. Vomiting	1 (2.1)	3 (6.1)
13. Sore throat	10 (20.8)	6 (12.2)
14. Cold/upper respiratory symptoms	10 (20.8)	14 (28.6)
15. Insomnia/interrupted sleep	16 (33.3)	14 (28.6)
16. Drowsiness	0 (0)	2 (4.1)
17. Fatigue	6 (12.5)	5 (10.2)
18. Agitation	13 (27.1)	11 (22.4)
19. Restless or fidgety	8 (16.7)	9 (18.4)
20. Disobedient/defiant	17 (35.4)	9 (18.4)
21. Anxiety/nervousness	31 (64.6)	22 (44.9)
22. Irritability	21 (43.8)	11 (22.4)
23. Emotional outbursts	24 (50)	14 (28.6)
24. Rage/aggressive outbursts	11 (22.9)	6 (12.2)
25. Hyperactivity	2 (4.2)	3 (6.1)
26. Disinhibition	2 (4.2)	2 (4.1)
27. Impulsivity	2 (4.2)	a. (0)
28. Difficulty thinking/confusion	3 (6.3)	5 (10.2)
29. Accidental injury	2 (4.2)	2 (4.1)
30. Skin rashes	4 (8.3)	a. (0)
31. Allergy	2 (4.2)	b. (0)
32. Asthma	2 (4.2)	c. (0)
33. Thoughts of killing self	0 (0)	1 (2)
34. Self-harm	0 (0)	1 (2)
35. Suicide attempt	0 (0)	a. (0)
36. Thoughts of killing someone else	0 (0)	0 (0)

CBT, cognitive behavioural therapy; SERT, sertraline; PBO, placebo.

*Note*: This table does not include the two children removed from the study due to suicidal ideation at session as these children did not receive medication.

**Fig. 2.**
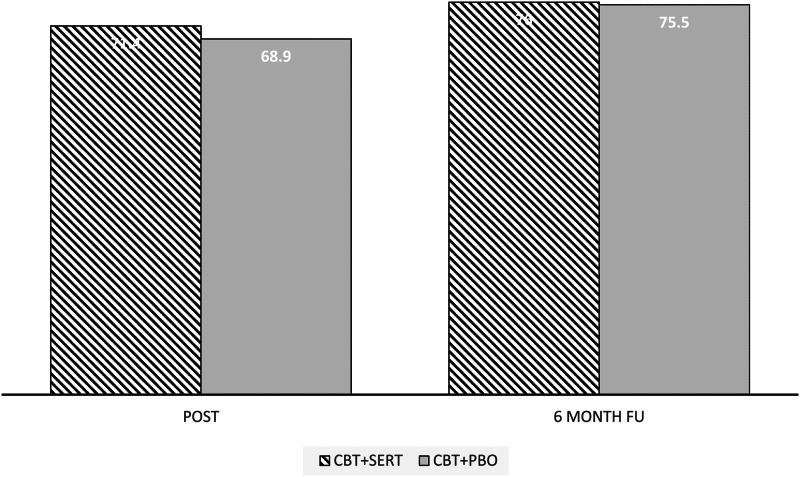
Remission rates of the primary anxiety disorder across condition and time.

**Fig. 3.**
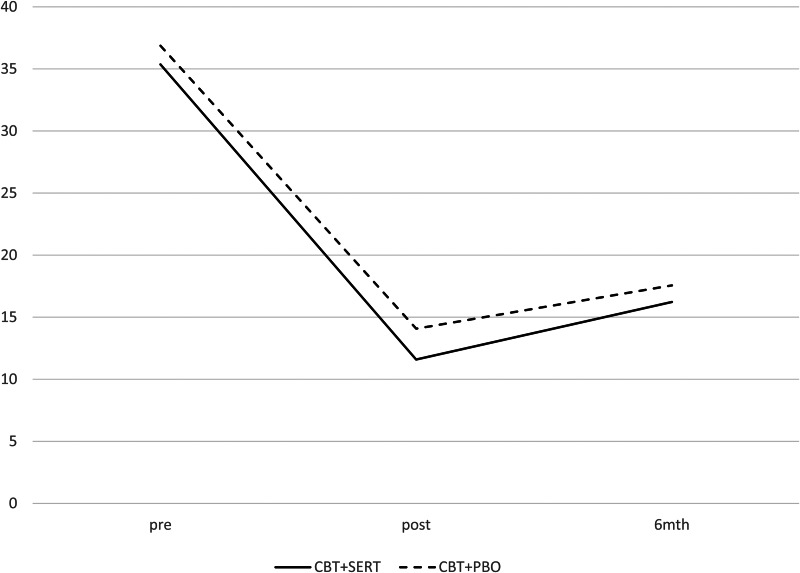
Primary care-giver-reported anxiety symptoms (SCAS) across condition and time.

The two conditions were compared on demographic and pre-treatment symptom measures as well as treatment credibility/expectations (see Tables 2 and 4) and no significant differences emerged (all *p*s > 0.05). At post and follow-up, there were no significant differences across conditions in remission rates based on primary disorder or all anxiety disorders (see Table 3 and Fig. 2). Mixed model analyses were fitted to CSRs, child-reported symptoms and mother-reported symptoms. Table 4 provides the descriptive statistics for the outcome variables and the slopes and intercepts for the models, respectively. There was a significant effect for time for each measure but no significant effect for condition or time × condition for clinical severity, anxiety symptoms (child and primary caregiver report), depression symptoms (child and primary caregiver report) and life interference (child and primary caregiver report) (see Table 4 and Fig. 3).

**Table 2.** Demographic and treatment credibility/expectancy data across conditions (% in parentheses)

Demographic	CBT + SERT (*N* = 50)	CBT + PBO (*N* = 49)
Gender male	23 (46.0)	31 (63.3)
Age in years (s.d.)	9.60 (1.94)	9.57 (2.18)
Family setting %two parent	44 (88)	41 (85.4)
Family income^1^		
>$208 000	14 (28)	7 (14.7)
$124 800–207 999	17 (36)	19 (39.6)
$8400–124 799	11 (22)	14 (29.2)
$0–83 999	7 (14)	8 (16.7)
Ethnicity^2^		
Australian	38 (76)	29 (58.3)
Asian/Asian Australian	3 (6)	0 (0)
European/European Australian	6 (12)	17 (35.4)
Other	3 (6)	3 (6.3)
Primary anxiety diagnoses		
GAD	21 (42.0)	27 (55.1)
SOC	12 (24)	11 (22.4)
SAD	8 (16)	6 (12.2)
PD	1 (2)	1 (2)
OCD	3 (6)	0 (0)
SPEC	4 (8)	1 (2)
OSAD	0 (0)	3 (6.1)
PTSD	1 (2)	0 (0)
Comorbid diagnoses		
Anxiety	41 (82)	42 (85.7)
Externalising	6 (12)	12 (24.5)
Mood disorders	2 (4)	1 (2)
Treatment credibility		
Parent report	*N* = 40	*N* = 39
Pre-treatment CBT total	41.60 (6.23)	38.54 (6.26)
Pre-treatment medication total	31.79 (9.33)	31.28 (7.78)
	*N* = 39	*N* = 41
Post-treatment CBT total	42.77 (6.44)	41.61 (5.41)
Post-treatment medication total	29.05 (11.20)	26.78 (11.74)
Child report	*N* = 39	*N* = 38
Pre-treatment CBT total	37.05 (9.84)	34.11 (10.81)
Pre-treatment medication total	34.49 (11.17)	29.42 (12.49)
	*N* = 37	*N* = 40
Post-treatment CBT TOTAL	45.65 (6.78)	38.10 (10.59)
Post-treatment medication total	34.11 (15.60)	26.13 (13.21)

CBT, cognitive behavioural therapy; SERT, sertraline; PBO, placebo; GAD, generalised anxiety disorder; SOC, social phobia; SAD, separation anxiety disorder; PD, panic disorder; OCD, obsessive compulsive disorder; SPEC, specific phobia; OSAD, other specified anxiety disorder.

1 Income is reported in Australian Dollars

2 Parents are asked to record their ethnicity in an open-ended question. The majority of our clients identify as ‘Australian’. A smaller proportion of families identified with North-West, Southern or Eastern European (e.g. Italian, Croatian-Australian) or South-East, North-East, Southern or Central Asian (Thai, Chinese-Australian) heritage.

**Table 3.** Proportion of children no longer meeting criteria for the primary anxiety diagnosis and all anxiety diagnoses at post and follow-up across the two conditions

	Post-treatment			Follow-up		
	CBT + SERT	CBT + PBO		CBT + SERT	CBT + PBO	
	*N* (%) 49	*N* (%) 45	Pearson χ^2^	*N* (%) 46	*N* (%) 39	Pearson χ^2^
Primary	71.4	68.9	χ^2^(1, *N* = 94) = 0.07, *p* = 0.79, Cohen's *d* = 0.05	73.9	69.2	χ^2^(1, *N* = 85) = 0.23, *p* = 0.63, Cohen's *d* = 0.10
Anxiety diagnoses	61.2	51.1	χ^2^(1, *N* = 94) = 0.98, *p* = 0.32, Cohen's *d* = 0.21	65.2	61.5	χ^2^(1, *N* = 85) = 0.12, *p* = 0.73, Cohen's *d* = 0.08
CGI-I						
Very much improved	44.9	36.4	χ^2^(3, *N* = 94) = 0.999, *p* = 0.80	67.4	50	χ^2^(3, *N* = 85) = 0.12, *p* = 0.73
Much improved	32.7	40		17.4	27.5	
Minimally improved	16.3	18.2		13	17.5	
No change	6.1	4.5		2.2	5	

CGI, Clinical Global Impression-Improvement; CBT, cognitive behavioural therapy; SERT, sertraline; PBO, placebo.

**Table 4.** Mean pretreatment, posttreatment and follow-up data across the two conditions in the completer sample (s.e.s in parentheses)

	CBT + SERT			CBT + PBO			*F* and *t* values and effect sizes
	Pre	Post	Follow-up	Pre	Post	Follow-up	
Measure	*M* (s.e.)	*M* (s.e.)	*M* (s.e.)	*M* (s.e.)	*M* (s.e.)	*M* (s.e.)	
CSR (primary)	5.92 (0.21)^a^	2.69 (0.21)^b^	1.99 (0.24)^c^	6.06 (0.22)^a^	2.91 (0.22)^b^	2.03 (0.27)^c^	Time *F*_(__2, 177)_ = 197.78, *p* < 0.05; Condition (1, 99) = 0.41, *p* > 0.05; Condition × time (2, 177) = 0.08, *p* > 0.05. Cohen's *d* Post = 0.12, Follow-up = 0.02. Within-subjects effect size (pre to post: CBT + SERT *d* = 1.80; CBT + PBO *d* = 1.88; Pre to follow-up: CBT + SERT *d* = 2.12; CBT + PBO *d* = 2.17; Post to follow-up: CBT + SERT *d* = 0.40; CBT + PBO = 0.47).
SCAS-C	38.58 (2.13)^a^	24.21 (2.15)^b^	22.56 (2.17)^b^	36.83 (2.15)^a^	23.74 (2.21)^b^	21.13 (2.25)^b^	Time *F*_(__2, 173)_ = 54.32, *p* < 0.05; Condition (1, 92) = 0.26, *p* > 0.05; Condition × time (2, 172) = 0.08, *p* = > 0.05. Cohen's *d* post = −0.05, follow-up = −0.11. Within subjects effect size (pre to post: CBT + SERT *d* = 0.93; CBT + PBO *d* = 1.05; pre to follow-up: CBT + SERT *d* = 1.16; CBT + PBO *d* = 1.12; post to follow-up: CBT + SERT *d* = 0.15; CBT + PBO *d* = 0.16)
SCAS-P	35.36 (1.59)^a^	11.60 (1.61)^b^	16.23 (1.64)^c^	36.87 (1.61)^a^	14.08 (1.64)^b^	17.57 (1.69)^c^	Time *F*_(__2, 178)_ = 230.04, *p* < 0.05; Condition (1, 95) = 0.91, *p* > 0.05; Condition × time (2, 178) = 0.14, *p* > 0.87. Cohen's *d* post = 0.24, follow-up = 0.10. Within subjects effect size (pre to post: CBT + SERT *d* = 2.21; CBT + PBO *d* = 2.34; pre to follow-up: CBT + SERT *d* = 1.53; CBT + PBO *d* = 1.57; post to follow-up: CBT + SERT *d* = −0.48; CBT + PBO *d* = −0.28).
SMFQ-C	5.22 (0.66)^a^	3.77 (0.67)^ab^	3.96 (0.67)^abc^	6.67 (0.67)^a^	3.68 (0.69)^b^	3.86 (0.67)^bc^	Time *F*_(__2, 173)_ = 12.54, *p* < 0.05; Condition (1, 93) = 0.30, *p* > 0.05; Condition × time (2, 173) = 1.62, *p* > 0.05. Cohen's *d* post = −0.03, follow-up = −0.02. Within subjects effect size (pre to post: CBT + SERT *d* = 0.29; CBT + PBO *d* = 0.95; pre to follow-up: CBT + SERT *d* = 0.26; CBT + PBO *d* = 0.57; post to follow-up: CBT + SERT *d* = −0.05; CBT + PBO *d* = −0.07)
SMFQ-P	6.76 (0.58)^a^	2.30 (0.59)^b^	2.56 (0.61)^bc^	7.79 (0.59)^a^	2.94 (0.60)^b^	4.20 (0.62)^c^	Time *F*_(__2, 171)_ = 65.59, *p* < 0.05; Condition (1, 87) = 2.65, *p* > 0.05; Condition × time (2, 173) = 0.61, *p* > 0.05. Cohen's *d* post = 0.16, follow-up = 0.43. Within subjects effect size (pre to post: CBT + SERT *d* = 1.3; CBT + PBO *d* = 1.20; pre to follow-up: CBT + SERT *d* = 0.80; CBT + PBO *d* = 0.62; post to follow-up: CBT + SERT *d* = −0.11; CBT + PBO *d* = −0.31)
CALIS-C	13.71 (1.04)^a^	8.12 (1.05)^b^	7.12 (1.06)^bc^	11.84 (1.05)^a^	9.57 (1.09)^ab^	8.10 (1.11)^bc^	Time *F*_(__2, 178)_ = 16.16, *p* < 0.05; Condition (1, 93) = 0.03, p > 0.05; Condition × time (2, 178) = 1.81, *p* > 0.05. Cohen's *d* post = 0.20, follow-up = 0.15. Within subjects effect size (pre to post: CBT + SERT *d* = 0.80; CBT + PBO *d* = 0.31; pre to follow-up: CBT + SERT *d* = 0.86; CBT + PBO *d* = 0.45; post to follow-up: CBT + SERT *d* = 0.18; CBT + PBO *d* = 0.14)
CALIS-P	26.20 (1.45)^a^	13.39 (1.46)^b^	13.90 (1.50)^bc^	27.88 (1.47)^a^	16.45 (1.49)^ab^	15.81 (1.45)^bc^	Time *F*_(__2, 180)_ = 92.24, *p* < 0.05; Condition (1, 96) = 1.66, *p* > 0.05; Condition × time (2, 180) = 0.25, *p* > 0.05. Cohen's *d* post = 0.30, follow-up = 0.19. Within subjects effect size (pre to post: CBT + SERT *d* = 1.30; CBT + PBO *d* = 1.09; pre to follow-up: CBT + SERT *d* = 1.10; CBT + PBO *d* = 1.06; post to follow-up: CBT + SERT *d* = −0.05; CBT + PBO *d* = 0.04)

PRE, pretreatment; POST, posttreatment; primary CSR, clinical severity rating of the child's principal diagnosis; SCAS, Spence Children's Anxiety Scale; SCAS, Spence Children's Anxiety Scale; SMFQ, Short Mood and Feelings Questionnaire; CBT, cognitive behavioural therapy; C, child; P, primary caregiver; SERT, sertraline; PBO, placebo.

abcShared superscripts denote no significant difference between means following contrasts between groups at pre, post and follow-up and within groups between pre-post, pre-follow-up and post to follow-up; *p* < 0.05/3.

*Note*: Between-subjects effect sizes calculated using the estimated marginal means and the pooled s.d. Within-subjects effect sizes calculated using the estimated marginal means and the s.d. for the later time point.

## Discussion

Walkup and colleagues observed a distinct advantage of combining medication and CBT over either monotherapy, yet failed to adequately control for expectancy effects as well as number of treatment visits (Walkup et al., 2008). Before conclusions are made about the use of medication and CBT as best practice for the treatment of child anxiety disorders, comparisons of this approach against a matched pill placebo design are essential. The current study evaluated the efficacy of combining anti-depressant medication and psychological treatment for paediatric anxiety disorders against an appropriate placebo. Consistent with earlier pilot studies (Cohen et al., 2007; Storch et al., 2013), the results of the study failed to demonstrate a statistically significant difference in treatment response between children receiving CBT and SSRI compared to children receiving CBT and a pill-placebo. These effects were consistent across diagnostic, symptom and life interference measures, and across independent assessor, parent and child reports. The observed effect sizes on anxiety measures were extremely small. The largest effect between the two conditions (albeit non-significant) was observed for parent-reported depression symptoms at 6-month follow-up: parents of children in the CBT and medication group reported fewer (but not significantly fewer) child depression symptoms compared to the CBT and pill-placebo group (*g* = 0.43). The size of the effect for parent-report depression at post-treatment was extremely small, so too was the difference between child-reported depression at post and follow-up. Overall, the results of the study show no robust differences between the two treatment conditions on child anxiety or depression.

Compared to remission rates observed in previous trials of CBT for child anxiety, the current remission rates indicate that adding a medication (either placebo or active) to CBT led to stronger remission rates. Remission rates in a large comparable sample from our clinic (i.e. anxiety disordered children with comorbid anxiety disorders but without comorbid depression as in the current study) show 43.7% of children at post and 52% at follow-up no longer met criteria for the primary anxiety disorder. In the current trial, children receiving CBT along with a daily placebo pill showed much higher remission rates of 68% at post and 69% at follow-up. This considerable increase in apparent efficacy along with the lack of difference between conditions in the current trial, suggests that positive expectancies may increase the effects of CBT. This suggestion is consistent with a large literature demonstrating the power of placebo (Kaptchuk & Miller, 2015).

Importantly, children in the CBT and SERT condition did not evidence significantly greater risk of suicide or other negative side-effects compared to the CBT and placebo condition. Results of this study suggest that SSRI medication, at the recommended daily dose, is well tolerated but does not enhance treatment for children with anxiety disorders. Rather, the results of this study imply that if we improve client expectations of treatment, such as through explaining the likely success of the treatment, this may lead to significantly better outcomes for families. This could also potentially be achieved by strengthening treatment rationales or augmenting treatments through motivational interviewing techniques prior to commencement of treatment (Romano & Peters, 2016; Westra, Constantino, & Antony, 2016).

Although the current study followed the same dosage protocol as that reported in the CAMS trial, the average daily dose prescribed to children in the current study was considerably less. Consistent with the Therapeutic Guidelines Australia recommendations for the use of SERT in children (6 years or older), starting with 25 mg daily and increasing to 1.5 mg/kg daily depending on efficacy and tolerability, the average dose in the CAMS trial was 133.7 mg with some children receiving a dose as high as four times the recommended dose (Limited, 2013). Thus, the absence of findings between the two conditions in the current study may be a result of the lower dosage. If this was a plausible explanation of the absence of effects, then one would expect the remission rates in the current study to be considerably lower than those achieved in the CAMS study. In contrast, the post treatment remission rate for SAD, GAD and social anxiety diagnoses in the CBT and SERT condition was 68%^2^ compared to 68.3% in the CAMS trial. Thus, the absence of statistically significant differences between the two conditions is unlikely to be a result of lower dosage.

Sixty percent of children were excluded from the study because they did not meet study criteria or were not willing to receive medication. This exclusion rate is markedly higher than that in our previously published trials (45.1%) (Hudson et al., 2014) and hence limits the degree to which the findings can be generalised to all children presenting for treatment. The sample is also predominately white, middle class and conducted in a university clinic further limiting generalisability.

In conclusion, the findings of the current study suggest that adding an SSRI to CBT did not significantly enhance outcomes above the effects of CBT combined with pill-placebo. This does not indicate that combining CBT with medication (SSRI or placebo) is not a worthwhile endeavour, especially given the strong effects observed in this trial compared to previous non-medication trials within our clinic. These results imply that the mechanisms responsible for the enhanced outcome observed in Walkup et al. (2008) may be something other than the physiological effects of the medication. Further research is needed to improve our understanding of the mechanisms of change following intervention and improve our understanding of strategies that can impact both positively and negatively on parent and child expectancies.

## Footnotes

† The notes appear after the main text.

## Notes

1 Note this is a lower average daily dosage than that of Walkup et al. (2008).

2 This remission rate has been calculated to be directly comparable to the CAMS trial which did not include remission of all anxiety diagnoses at post treatment only remission of SAD, GAD and social anxiety disorder.

## Supplementary material

For supplementary material accompanying this paper visit https://doi.org/10.1017/S0033291721003329. click here to view supplementary material
