# Supplementary material for: Combining CBT and sertraline does not enhance outcomes for anxious youth: a double-blind randomised controlled trial
Source: Psychol Med. 2021 Aug 31;53(5):1741–9. doi: 10.1017/S0033291721003329 (PMC10106296; doi:10.1017/S0033291721003329)
Supplement: Supplementary file 1 [file S0033291721003329sup001.docx]

Supplementary

Table S1: Complete list of mild, to severe adverse effects across conditions

|  | CBT+PBO  (n = 48) | CBT+SERT  (n = 49) |
| --- | --- | --- |
| 1. Tremors | 5 (10.4) | 7 (14.3) |
| 1. Fever | 5 (10.4) | 4 (8.2) |
| 1. Headache | 15 (31.3) | 9 (18.4) |
| 1. Dizziness | 6 (12.5) | 8 (16.3) |
| 1. Body ache | 12 (25) | 12 (24.5) |
| 1. Weightloss/decreased appetite | 7 (14.6) | 6 (12.2) |
| 1. Stomach pain | 18 (37.5) | 18 (36.7) |
| 1. Gastric distress | 11 (22.9) | 10 (20.4) |
| 1. Constipation | 8 (16.7) | 7 (14.3) |
| 1. Diarrhoea | 9 (18.8) | 10 (20.4) |
| 1. Nausea | 12 (25.0) | 15 (30.6) |
| 1. Vomiting | 2 (4.2) | 7 (14.3) |
| 1. Sore Throat | 25 (52.1) | 18 (36.7) |
| 1. Cold/Upper Respiratory symptoms | 27 (56.3) | 26 (53.1) |
| 1. Insomnia/interrupted sleep | 24 (50) | 25 (51) |
| 1. Drowsiness | 5 (10.4) | 9 (18.4) |
| 1. Fatigue | 13 (27.1) | 15 (30.6) |
| 1. Agitation | 25 (52.1) | 23 (46.9) |
| 1. Restless or fidgety | 21 (43.8) | 20 (40.8) |
| 1. Disobedient/defiant | 28 (58.3) | 22 (44.9) |
| 1. Anxiety/nervousness | 40 (83.3) | 33 (67.3) |
| 1. Irritability | 34 (70.8) | 28 (57.1) |
| 1. Emotional Outbursts | 35 (72.9) | 28 (57.1) |
| 1. Rage/Aggressive outbursts | 19 (39.6) | 12 (24.5) |
| 1. Hyperactivity | 8 (16.7) | 9 (18.4) |
| 1. Disinhibition | 8 (16.7) | 7 (14.3) |
| 1. Impulsivity | 5 (10.4) | 5 (10.2) |
| 1. Difficulty thinking/confusion | 10 (20.8) | 10 (20.4) |
| 1. Accidental injury | 3 (6.3) | 7 (14.3) |
| 1. Skin rashes | 7 (14.6) | 5 (10.2) |
| 1. Allergy | 5 (10.4) | 3 (6.1) |
| 1. Asthma | 4 (8.3) | 2 (4.1) |
| 1. Thoughts of killing self | 2 (4.2) | 3 (6.1) |
| 1. Self harm | 0 (0) | 1 (2) |
| 1. Suicide attempt | 0 (0) | 0 (0) |
| 1. Thoughts of killing someone else | 0(0) | 1 (2) |

Note: This table does not include the two children removed from the study due to suicidal ideation at session as these children did not receive medication.
